# Phthalate plasticizer affects blood electrolytes, hormones, and reproductive parameters of black Bengal goats

**DOI:** 10.5455/javar.2024.k856

**Published:** 2024-12-29

**Authors:** Sajibul Hasan, Afrina Mustari, Kazi Rafiq, Mohammad Alam Miah

**Affiliations:** 1 Department of Physiology, Faculty of Veterinary Science, Bangladesh Agricultural University, Mymensingh, Bangladesh; 2 Department of Pharmacology, Faculty of Veterinary Science, Bangladesh Agricultural University, Mymensingh, Bangladesh

**Keywords:** Phthalates, exposure, hormone, electrolyte, reproduction, BBG

## Abstract

**Objective:**

The present study looked at how electrolytes, hormones, and postpartum reproductive physiology were affected in black Bengal goats (BBGs) when they were fed a phthalate mixture (PHA).

**Materials and Methods:**

Twenty clinically healthy BBGs, 1–2 months pregnant, aged 6–8 months with a body weight of 10–12 kg, were chosen and randomly allocated to two experimental groups (*n = *10 each). The BBGs of the treatment group (*n = *10) were administered a standard ration containing a mixture of Diethyl Phthalate, Dibutyl Phthalate, Di-isobutyl Phthalate, and Dipropyl Phthalate phthalate. The non-treated control group (*n = *10) received the goat ration without any phthalates up to parturition. Blood samples were taken from experimental pregnant goats just before parturition to analyze serum hormone and electrolyte levels.

**Results:**

The levels of sodium, chloride, and calcium were reduced (*p *< 0.05) in PHA-exposed goats than in the non-exposed control group. No significant difference was observed in potassium and phosphorus levels between the two groups. PHA-exposed goats showed significantly decreased levels of estrogen, progesterone, luteinizing hormone, and thyroxin compared to control goats (*p *< 0.05). Postpartum reproductive traits, such as gestation length, postpartum heat period, abortion rate, and retained placenta, were significantly (*p *< 0.05) prolonged in BBG that had been exposed to PHA.

**Conclusion:**

PHA plasticizer exposure during pregnancy affects the electrolytes, hormones, and postpartum reproductive physiology of BBGs.

## Introduction

Phthalates (PHA) are ubiquitous in our environment. PHA has been frequently used in industries, notably as plasticizers in diverse applications. They have shown deleterious effects in people and laboratory animals by functioning as an endocrine disruptor [1]. PHA is extensively utilized in popular consumer products such as materials for plastics, skincare products, toiletries, toys for kids, vinyl flooring, plastic devices for medicine, processed food items, pesticide products, and high-fat dairies [2].

The widespread nature of these chemicals consistently exposes animals via food, inspiration, and skin contact. PHA contamination is considered a widespread and pervasive public health issue due to its constant presence in food, water, and air, and its absorption through the skin. Approximately 7.5 million tons of plasticizers are utilized annually in specific sectors, including construction products, leather products, healthcare equipment, scents, and cosmetic products [3]. Reproductive disorders are the most notable disorders linked to PHA. There is a global phenomenon of rising rates of reproductive disorders, including hormone-dependent cancers, infertility, and reduced fertility [4]. One of the most significant impacts of PHA is its effect on fetal development and the occurrence of abnormalities in conception, which are commonly known as “phthalate conditions” [5]. The effects of PHA are inevitable at any stage of life, but the fetal, perinatal, and early infancy phases are the most susceptible, as they have a lasting impact on physiological parameters [6].

PHA causes pregnancy complications, such as extended or shortened pregnancy, impaired growth of blood vessels in the placenta, and abortion through alteration of PPAR and prostaglandin functions [7]. PHA affects both pregnancy outcomes and cancer development in female reproductive organs. PHA can disrupt the HPG axis at various regulatory levels, causing reproductive disorders. At the hormonal level, PHA has an impact on steroid-producing enzymes, hormones, and sex hormone-binding globulin [8]. PHAs are substances that can harm reproductive organs and interfere with ovarian follicles’ normal development, growth, and hormone production [9].

PHA can cause irregular menstrual or estrous cycles, decrease fertility, or lead to infertility. The impact of PHA on the regularity of menstrual cycles in women is not thoroughly understood. However, studies on animals have demonstrated that phthalate exposure disrupts the regularity of estrous cycles [10]. PHA can have a negative effect on the developing ovaries, causing problems with the ovarian follicle reserve, messed up folliculogenesis, lower levels of gonadotropins, and the growth of ovarian lesions in women [11]. Any interruption in the development of the initial pool of primordial follicles can impact a woman’s reproductive capacity. PHA disrupts the hormonal regulation and normal functioning of the endocrine system, thereby impacting the well-being and reproductive abilities of humans as well as animals [12].

PHA impacts the activity of the hypothalamic-­hypophyseal-gonadal axis at the hormonal level. This axis plays a vital role in ensuring the appropriate development of the reproductive system both before and after birth. Reproductive disorders may arise if there is an inadequate or excessive amount of sex hormones. These chemicals can affect the development and function of the hormone-­dependent portions of the reproductive system [13]. PHA has anti-­estrogenic effects that stop the ovary from making estradiol, which leads to anovulation and early ovarian insufficiency. Phthalate exposure is linked to reduced estradiol levels, lower fertility, and anovulation. No studies have been reported regarding the impact of these environmental contaminants on the fertility parameters of small ruminants. BB goats possess the capacity to significantly enhance economic stability and nutritional security for disadvantaged individuals. This, therefore, results in a comprehensive enhancement of Bangladesh’s gross domestic product [14]. We hope that this research will help to find out the baseline of electrolytes, hormonal, and reproductive data in goats treated with PHA significantly and, thereby, contribute to improving the sustainable productivity of goats in Bangladesh.

## Materials and Methods

### Ethical approval

The Animal Welfare and Experimentation Ethics Committee of Bangladesh Agricultural University, Mymensingh, approved the present study [AWEEC/BAU/2020-20], and all experimental procedures were conducted in accordance with their guidelines for the care and use of animals.

### Experimental designs

Twenty clinically sound, 1–2 months pregnant Black Bengal (*Capra hircus*) goats (BBG), weighing 10–12 kg and aged between 6 and 8 months, were chosen for this study. The pregnant BBGs were randomly allocated into two experimental groups, each consisting of 10 goats. The control group was given a standard goat ration with corn oil. The treatment group was given rations containing PHA. The PHA was given at 100 mg/kg of BW daily for 100 days of the experimental period. Throughout the study period, procedures for management in the experimental shed were maintained with maximal uniformity.

### Experimental chemicals

Diethyl Phthalate, Dibutyl Phthalate (DBP), Di-isobutyl Phthalate (DIBP), and Dipropyl Phthalate were procured from Sigma-Aldrich Company, Germany, for the experiment. A pure phthalate mixture was produced by calculating and combining the appropriate quantity of each phthalate in accordance with the protocol outlined in [15]. Before administration, these chemicals were dissolved in corn oil (vehicle) as a stock solution. PHA was diligently managed to prevent any potential exposure.

### Collection of blood samples

Around 5 ml of blood samples were aseptically drawn from the jugular vein of each goat and transferred into a vacutainer tube for serum separation, facilitating subsequent analysis of electrolytes and hormones.

### Analysis of electrolytes and minerals

The electrolyte parameters, including i) sodium (mmol/l), ii) potassium (mmol/l), and iii) chloride (mmol/l), were analyzed using the automated electrolyte analyzer Vitros-250 (J&J)/Dade Behring Dimension RxL, and the mineral parameters like i) calcium (mg/dl) and ii) phosphorus (mg/dl) were analyzed by an automated biochemical analyzer (Vitros-5600 (J&J)/Beckman Coulter AU480) using the electrolyte and minerals reagent kits (Sodium-Cat. No: 8.05.34.0.0050; Potassium-Cat. No: 8.05.33.0.0050; Chloride-Cat. No: 8.05.10.0.0250; Calcium-Cat. No: 8.05.08.0.0250; Phosphorus-Cat. No: 8.05.32.0.0250) provided by the automated biochemical analyzer (Vitros -5600 (J&J)/Beckman Coulter AU480) using the electrolyte and minerals reagent kits (Sodium- Cat. No: 8.05.34.0.0050; Potassium-Cat. No: 8.05.33.0.0050; Chloride-Cat. No: 8.05.10.0.0250; Calcium-Cat. No: 8.05.08.0.0250; Phosphorus–Cat. No: 8.05.32.0.0250) provided by Atlas Medical Company, Germany.

### Analysis of hormones

The hormones including- i) serum estrogen (pg/ml), ii) progesterone (ng/ml), iii) luteinizing hormone (mIU/ml), iv) serum thyroxine (ug/ml), and v) growth hormone (ng/ml) were determined by ELISA method using the specific reagents (Estrogen hormone- Cat No: CSB-E13505G; Progesterone Hormone-Cat No: CSB-E08172b; Luteinizing Hormone-Cat No: CSB-E13274G) provided by Atlas Medical Company, Germany using automated hormone analyzer machine Vitros -5600 (J&J)/Abbott Architect I -1000-SR.

### Analysis of reproductive traits

The gestation length of the BBGs was determined by measuring the time from the servicing period to the date of parturition. The litter size was calculated based on the number of kids born per goat after parturition. The post-partum heat period refers to the duration during which goats exhibit their initial estrous cycle after giving birth. The abortion rates were calculated by the number of induced abortions occurring in a specified experimental period. The retained placenta was estimated by the time when it had not passed within 12 h after the parturition of the experimental goats.

### Statistical analysis

The acquired data was imported and saved into the Excel spreadsheet. Subsequently, the data was examined using GraphPad Prism software (V8.0) and then subjected to a student-paired *t*-test. The results are presented as the mean value plus or minus the standard error of the values of the collected data from the assigned experimental goats three times (15-day intervals); each replication/time contained at least three goats. A probability (*p*) value below 0.05 counts as statistically significant.

## Results and Discussion

### Impact of PHA on electrolytes in BBGs

Table 1 shows the effect of PHA on electrolytes in BBGs. During pregnancy, the goats exposed to PHA in their diet had significantly lower levels of sodium and chloride than the non-treated control group (*p* < 0.05). The calcium levels in PHA-exposed goats were significantly lower (*p *< 0.05) compared to the non-treated control goats (Table 1). The levels of potassium and phosphorus were within the normal range. Plasticizer exposure can cause symptoms in ruminants, including reduced appetite, lack of water, recurring bloating, and deficiencies in minerals and vitamins. If not treated promptly, these animals may ultimately die [16]. When these chemicals infiltrate the body’s biological tissues, such as rumen fluid, milk, blood, flesh, and eggs, they can cause numerous harmful endocrine disorders [17].

**Table 1.** Electrolyte values (mean ± SE) of BBG exposed to environmentally relevant PHA.

Electrolytes and minerals	Control goats	PHA exposed goats
Sodium (mg/dl)	143.40 ± 0.68	138.40 ± 1.44*
Potassium (mg/dl)	5.70 ± 0.14	5.04 ± 0.18 ns
Chloride (mg/dl)	109.00 ± 0.45	102.20 ± 0.86*
Calcium (mg/dl)	9.64 ± 0.09	7.66 ± 0.52*
Phosphorus (mg/dl)	7.45 ± 0.13	7.28 ± 0.22 ns

* Differ significantly at *p* < 0.05 (normal *vs.* PHA exposed goats); ns-not-significant.

Additionally, they can lead to changes in various hematological and biochemical parameters, as well as electrolyte concentrations [18]. A study by Kumar et al. [19] found that animals exposed to plasticizers had serum sodium levels within the normal range for their species. Nevertheless, hyponatremia in animals exposed to PHA as a result of the malfunctioning of the sodium ionic pump, which corroborates our study. Hypochloremia typically occurs in cases of gastrointestinal tract obstruction, where the blockage prevents the omasum and abomasum from emptying, leading to an accumulation of chloride ions in the rumen. Similarly, reports of [19] found that cattle with ruminal plastic impaction exhibited hypochloremia. Over time, cattle exposed to plasticizers and developing plastic impaction experienced mildly low potassium levels in their blood due to dehydration and prolonged loss of appetite [20]. Researchers have reported similar findings [19]. Atonic or a hypotonic ruminal condition, inadequate nutrition, prolonged anorexia, and alkalosis cause the failure of calcium absorption through the gut, leading to low calcium levels in plasticizer-exposed cattle [21]. The results were consistent with our current study. Consistent with our findings, [19] also reported no alteration in phosphorus concentrations. According to [22], hypophosphatemia has been reported in cattle with rumen plastic impaction.

### Impact of PHA on hormones in BBGs

**Figure 1.**
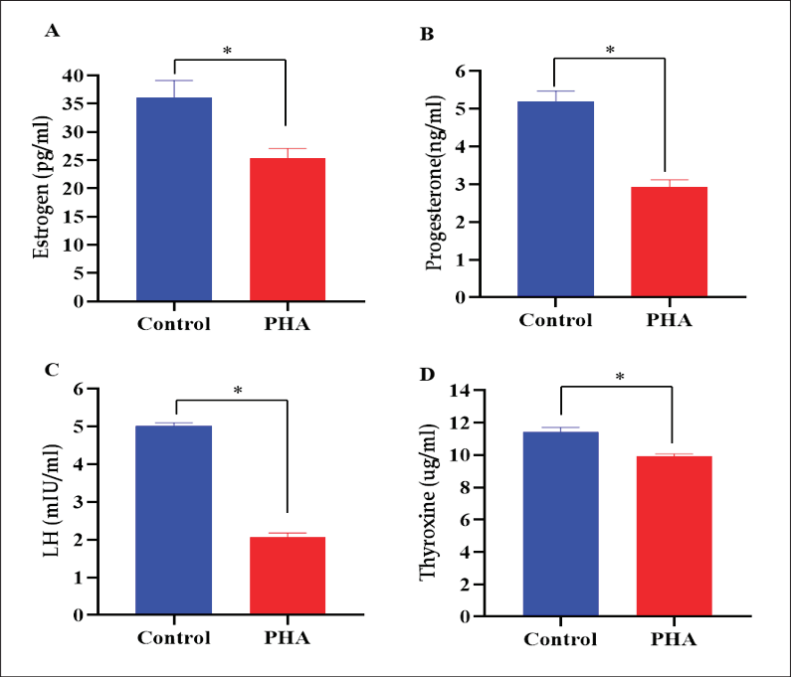
Reproductive hormone levels (mean ± SE) of BBG exposed to environmentally relevant PHA. *Differ significantly at *p *< 0.05 (Normal *vs.* PHA exposed BBGs).

The levels of estrogen, progesterone, luteinizing hormone, and thyroxin (T4) hormone were significantly (*p *< 0.05) reduced in PHA-exposed BBGs compared to the levels observed in control goats (Fig. 1). Phthalate exposure might disrupt the maternal hypothalamic-­hypophyseal-thyroid axis. The present findings are in line with those of [23], which showed that PHA exposure markedly decreased estradiol synthesis in cultured antral follicles in mice and inhibited steroidogenesis. PHA exhibits anti-­estrogenic activity by suppressing estradiol synthesis in the ovary, resulting in anovulation and early ovarian insufficiency [24].

Maternal exposure to PHA affects the response of progesterone due to a change in progesterone receptor gene expression levels. So phthalate exposure may lead to adverse pregnancy outcomes via disruption of progesterone concentrations throughout pregnancy. Phthalate exposure significantly decreased the expression of CYP17A1 and luteinizing hormone choriogonadotropin receptor in thecal cells, leading to reduced levels of progesterone, estradiol, androstenedione, and LH [25] and is associated with thyroid and growth hormone parameters. They may reduce the production of sodium-iodine cotransport proteins or have negative impacts on T4 or T3 hormones, resulting in reduced thyroxine levels [26], which are agreed to in our study [27]. They stated that phthalate exposure can disrupt thyroid-stimulating hormone (TSH) signaling by interacting with TSH receptors, binding to transporter proteins, and modifying the uptake of iodine by thyroid follicular cells through various potential processes. PHA is linked to modifications in thyroid, progesterone, and estrogen function in both pregnant and non-pregnant individuals [28].

### Impact of PHA on post-partum reproductive parameters in BBGs

The reproductive parameters, including gestation length, post-partum heat period, abortion rate, and retained placenta of BBGs, are presented in Table 2. These parameters were significantly (*p *< 0.05) altered in goats exposed to PHA, but the litter size didn’t differ significantly (*p* > 0.05) from the control goats. PHA affects the length and process of pregnancy. Bisphenol A and PHA adversely affect reproduction, the thyroid gland, and the immune system in humans and animals. PHA causes pregnancy dysfunctions, including extended or shortened gestation and abortion, through the modulation of PPAR and prostaglandin action. The present findings align with the findings of [29], who reported that PHA and its metabolites have been linked to a variety of negative perinatal outcomes, including premature birth, impaired neurodevelopment, and asthma, due to their impact on placental development and function. PHA increased the release of prostaglandins, which could lead to spontaneous abortion or premature delivery.

**Table 2.** Reproductive parameters (mean ± SE) of BBGs exposed to PHA.

Reproductive parameters	Control goats	PHA exposed goats
Gestation length (days)	152.00 ± 1.38	164.00 ± 1.87*
Litter size (number)	2.00 ± 0.20	1.60 ± 0.24^ ns^
Post-partum heat period (days)	35.00 ± 1.84	92.40 ± 3.04*
Abortion rate (%)	0.50 ± 0.35	32.00 ± 0.71*
Retained placenta (%)	0.00 ± 0.71	35.00 ± 0.71*

* Values in a row of the table differ significantly at *p *< 0.05 (normal *vs.* Phthalates exposed goats.

The signaling molecules known as prostaglandins cause the uterus to contract, which can result in either a baby being born or an abortion. Administering DBP at doses of 500 and 1000 mg/kg/day from weaning through puberty, mating, and gestation resulted in mid-pregnancy abortion and a decreased proportion of live pups delivered in rats. [30]. Animal pregnancy duration is negatively impacted by phthalate exposure, and postnatally hypodermically injecting DEHP into female mice reduced the weights of F1 pups and the number of their litters. The current findings align with [29,30], which indicated that PHA affects pregnancy and fetal development, including links to premature birth. These effects may arise from the disruption of endocrine pathways or through mechanisms such as oxidative stress and/or inflammation.

## Conclusion

The present research expresses that gestational exposure to PHA markedly influences the electrolyte, endocrine, and reproductive parameters of BBGs. Financial losses occur not only because of reproductive and hormonal failures but also because of the cost of medication and labor required to treat and care for sick goats. Nevertheless, the data produced in the present study could serve as valuable reference points for the scientific community regarding BBGs exposed to PHA. We hope that these studies may significantly contribute to finding out the causes of hormonal, electrolyte, and reproductive problems and, thereby, improve the sustainable productivity of goats in Bangladesh.

## List of abbreviations

BBGs, Black Bengal goats; DBP, Dibutyl phthalate; EDCs, Endocrine disrupting chemicals; PHA, phthalate.
